
JOURNAL INFORMATION
==============================
NLM Title Abbreviation: Cytotherapy
Journal ID: Cytotherapy
NLM Journal ID: 100895309

Cytotherapy

ISSN: 1465-3249
EISSN: 1477-2566

ARTICLE INFORMATION
==============================
PMCID: PMC10581616
Article ID: NIHMS1912171
PMID: 37517864
DOI: 10.1016/j.jcyt.2023.06.001
Article ID: NIHMS1912171
Article ID: NIHPA1912171
Article version: 1
Subjects: Article

Corrigendum to ABCB5+ mesenchymal stromal cells facilitate complete and durable wound closure in recessive dystrophic epidermolysis bullosa [Cytotherapy 25 (2023) 782–788/1562]

Dieter Kathrin 1 *
Niebergall-Roth Elke 2 *
Daniele Cristina 1
Fluhr Silvia 1
Frank Natasha Y. 3 4 5 6
Ganss Christoph 1 2
Kiritsi Dimitra 7
McGrath John A. 8
Tolar Jakub 9
Frank Markus H. 5 6 10 11 **
Kluth Mark A. 1 2 ** ***
1 RHEACELL GmbH & Co. KG, Heidelberg, Germany
2 TICEBA GmbH, Heidelberg, Germany
3 Department of Medicine, VA Boston Healthcare System, Boston, Massachusetts, USA
4 Division of Genetics, Brigham and Women’s Hospital, Harvard Medical School, Boston, Massachusetts, USA
5 Transplant Research Program, Boston Children’s Hospital, Harvard Medical School, Boston, Massachusetts, USA
6 Harvard Stem Cell Institute, Harvard University, Cambridge, Massachusetts, USA
7 Department of Dermatology, Medical Center – University of Freiburg, Faculty of Medicine, Freiburg, Germany
8 St John’s Institute of Dermatology, Guy’s Hospital, King’s College London, London, UK
9 Division of Blood and Marrow Transplantation and Cellular Therapy, Department of Pediatrics, University of Minnesota M Health Fairview Masonic Children’s Hospital, Minneapolis, Minnesota, USA
10 Department of Dermatology, Brigham and Women’s Hospital, Harvard Medical School, Boston, Massachusetts, USA
11 School of Medical and Health Sciences, Edith Cowan University, Perth, Western Australia, Australia
* These authors contributed equally to this work and share first authorship.

** These authors contributed equally to this work and share senior authorship.

*** Correspondence: Dr. Mark Andreas Kluth, TICEBA GmbH, Im Neuenheimer Feld 517, 69120 Heidelberg, Germany. andreas.kluth@ticeba.com (M.A. Kluth).
Print publication date: 2023 Sep
Volume: 25
Issue: 9
First page: 1016
Last page: 1016
Author manuscript; available in PMC: 2023 Oct 17
License: This is an open access article under the CC BY license (http://creativecommons.org/licenses/by/4.0/)
License URL: https://creativecommons.org/licenses/by/4.0/

Erratum for: ABCB5+ mesenchymal stromal cells facilitate complete and durable wound closure in recessive dystrophic epidermolysis bullosa 25 7 1 3 2023 782 788 Cytotherapy PMC10257763 36868990

==============================
The authors regret that the following funding information was missed: Contributions by JT to this work were supported by National Institutes of Health (NIH), National Institute of Arthritis and Musculoskeletal and Skin Diseases (NIAMS) grant #R01AR063070. The authors would like to apologize for any inconvenience caused.

